# Corrigendum to “Integration of datasets to provide insights about households’ natural gas expenditure as trigger to building stock decarbonisation” [Heliyon 9(4) (April 2023) e14922]

**DOI:** 10.1016/j.heliyon.2023.e16296

**Published:** 2023-05-19

**Authors:** Iná E.N. Maia, R.M. Moraes, R.T. Almeida, L. Kranzl, A. Müller, F. Schipfer

**Affiliations:** a Technische Universität Wien, Austria; b Universidade Federal do Rio de Janeiro and Instituto Brasileiro de Geografia e Estatística, Brazil; c Universidade Federal do Rio de Janeiro, Brazil; d e-think, Austria

In the original published version of this article, the authors have not provided the complete funding information. The funding information has now been updated. *The correct version of* funding *information can be found below*:

The authors acknowledge 10.13039/501100012650TU Wien Bibliothek for financial support through its Open Access Funding Program.

The authors apologize for the errors. Both the HTML and PDF versions of the article have been updated to correct the errors.

## Declaration of competing interest

The authors declare no conflict of interest.

